# The Eleemosynary Institution of Texas and the Governor’s Appointments

**Published:** 1903-01

**Authors:** 


### Abstracts and Selections.

### The Eleemosynary Institution of Texas and the Governor's Appointments.

Dr. M. K. Lott, of Cameron, Texas, while in Galveston recently, was interviewed by a representative of the *Galveston News,* and the following very sensible and broad-gauged statement appeared in a recent issue of that paper:

"Now that Governor-elect Lanham has announced the names of those who are to superintend several of the eleemosynary institutions as well as that of the State Health Officer, the medical profession of the State is gratified with the sound policy he pursued in retaining the present superintendents of the insane, the deaf and dumb and blind asylums, as well as the State Health Officer.

"The medical profession well knows how very difficult it is to thoroughly master these branches, and how meager the pecuniary reward to the average physician who undertakes to perform this service.

"The various forms of insanity are the most intricate and complex of all, requiring a fitness only to be obtained by much study and experience at the bedside.

"Special study and training are necessary to qualify those who are to intelligently treat the insane. The State owes it to herself and to humanity as well as to this most unfortunate class of her wards to provide for them the most enlightened and advanced treatment known to the profession. This is not to be had in the person of some political doctor, who was able to carry his ward for the administration at the primary or the polls. The helpless insane are turned over to him as his part of the spoils. Those of us who have lived long in Texas could name more than one such appointment to the superintendency of the insane asylums who could not have classified diseases of the mind, much less properly manage such an institution.

"If I mistake not, the superintendent of the insane asylum at Austin has been employed as such for the past twelve years. There is scarcely a physician or even a citizen of the State who is not cognizant of the splendid work he is doing in this field, guided by his great ability acquired largely during the incumbency of his present position. He is not a friend of mine; scarcely an acquaintance. The profession has every reason to believe that whatever is said of Dr. Worsham will be equally true of Dr. Graves of the asylum at San Antonio.

"We feel that the insane are very fortunate, and that Governor Lanham and the State are to be congratulated on the wisdom he has exercised in leaving the State Health Officer, whose enthusiasm for and hard study of quarantine have rendered him extraordinarily proficient as the chief guardian of our health. So with the other heads of the institutions mentioned.

"For the next four years then, should Governor Lanham live, we believe these eleemosynaries of the State are blessed with competent managers, but after this, then what? In order to protect these helpless wards of the State from the spoilsman in future administrations I would suggest that a law be passed to make the office of superintendent of the asylums for the deaf and dumb, the blind and the insane, if not others, for life or efficiency, with a salary of $5,000 a year, and in order that in their selection no mistake be made in getting the very best and most competent physicians for the service for which he is selected the applicant stand a competitive examination by the faculty of the medical department of the University of Texas here as to professional qualifications for the place, after satisfying them of moral and other necessary elements of character.

"Such superintendents securing their positions by such methods, and for life and a $5,000 salary attached, you may be assured will bring the very best in the United States as applicants for these positions, and once secured and for life, the appointee begins to more thoroughly fortify himself for the duties of his profession by availing himself of every opportunity offered, such as new books, new methods and attendance on associations of superintendents of such institutions, national and international.

"My remarks in this connection also apply to the epileptic asylum soon to be established at Abilene. The plans and specifications of the institution have been made public.

"It seems to me that the proper care of fifteen hundred patients, sick in mind and body, is quite enough labor for one man, however competent he may be, without requiring him to manage the physical property of such a large institution as the Austin asylum, which has, I am told, six hundred acres in pasture, etc., and the products of the dairy are no inconsiderable part of the output, and he has to look after all of this himself. He also has to give attention to the purchasing of supplies, to stock, killing and curing of meats, the keeping of accounts, and a multitude of such duties.

"These could well be committed to the care of a practical farmer, whose position should be superintendent of such and such asylum, his appointment being by the Governor, who doubtless would be well qualified for the position. I also think that the title of the medical director should be physician to the insane of such and such an asylum."

